# Drug and Chemical Allergy: A Role for a Specific Naive T-Cell Repertoire?

**DOI:** 10.3389/fimmu.2021.653102

**Published:** 2021-06-29

**Authors:** Rami Bechara, Alexia Feray, Marc Pallardy

**Affiliations:** ^1^ Division of Rheumatology & Clinical Immunology, University of Pittsburgh, Pittsburgh, PA, United States; ^2^ Inflammation, Microbiome and Immunosurveillance, Université Paris-Saclay, INSERM, Châtenay-Malabry, France

**Keywords:** naive T cells, drug allergy, hapten, thymic selection, TCR

## Abstract

Allergic reactions to drugs and chemicals are mediated by an adaptive immune response involving specific T cells. During thymic selection, T cells that have not yet encountered their cognate antigen are considered naive T cells. Due to the artificial nature of drug/chemical-T-cell epitopes, it is not clear whether thymic selection of drug/chemical-specific T cells is a common phenomenon or remains limited to few donors or simply does not exist, suggesting T-cell receptor (TCR) cross-reactivity with other antigens. Selection of drug/chemical-specific T cells could be a relatively rare event accounting for the low occurrence of drug allergy. On the other hand, a large T-cell repertoire found in multiple donors would underline the potential of a drug/chemical to be recognized by many donors. Recent observations raise the hypothesis that not only the drug/chemical, but also parts of the haptenated protein or peptides may constitute the important structural determinants for antigen recognition by the TCR. These observations may also suggest that in the case of drug/chemical allergy, the T-cell repertoire results from particular properties of certain TCR to recognize hapten-modified peptides without need for previous thymic selection. The aim of this review is to address the existence and the role of a naive T-cell repertoire in drug and chemical allergy. Understanding this role has the potential to reveal efficient strategies not only for allergy diagnosis but also for prediction of the immunogenic potential of new chemicals.

## Introduction

Adverse drug reactions (ADRs) are a major public health problem. Up to one third of ADRs are attributable to unpredictable drug hypersensitivity mediated by an adaptive immune response and named drug allergy. The consequences of drug and chemical allergy can be severe, including systemic adverse effects (1–3). T cells are central to allergic reactions. On one hand, drug-specific T cells provide the necessary help for mounting an effective B-cell response observed in immediate-type hypersensitivity reactions. On the other hand, T cells constitute the main pathogenic effector cells in delayed hypersensitivity reactions (4–6). Most studies have focused on the identification of memory T cells that recognize drugs/chemicals and the insights obtained have led to the development of allergy diagnostic tests (7–21). However, attention has recently turned to the naive T-cell repertoire, since it may largely determine the efficacy of the induced immune response (22).

The aim of this review is to describe the role of the naive T-cell repertoire in drug and chemical allergy. We provide an overview of the data supporting different models of T-cell recognition of drugs and chemicals and discuss speculative models addressing the origin of drug/chemical responsive naive T cells.

## Notion of Naive T Cell Repertoire

The identification of lymphocytes as the main cell type responsible for both cellular and humoral immunity started in the early 1950s with the emergence of cell culture techniques. It is now clear that the ability of T cells to promote an effective immune response depends on a large repertoire of unique T-cell receptors (TCRs) generated and selected in the thymus. Indeed, T-cell precursors randomly and imprecisely rearrange V and J segments of the TCR alpha and V, D, and J segments of the TCR beta chains to create a complete TCR.

**Figure 1:**
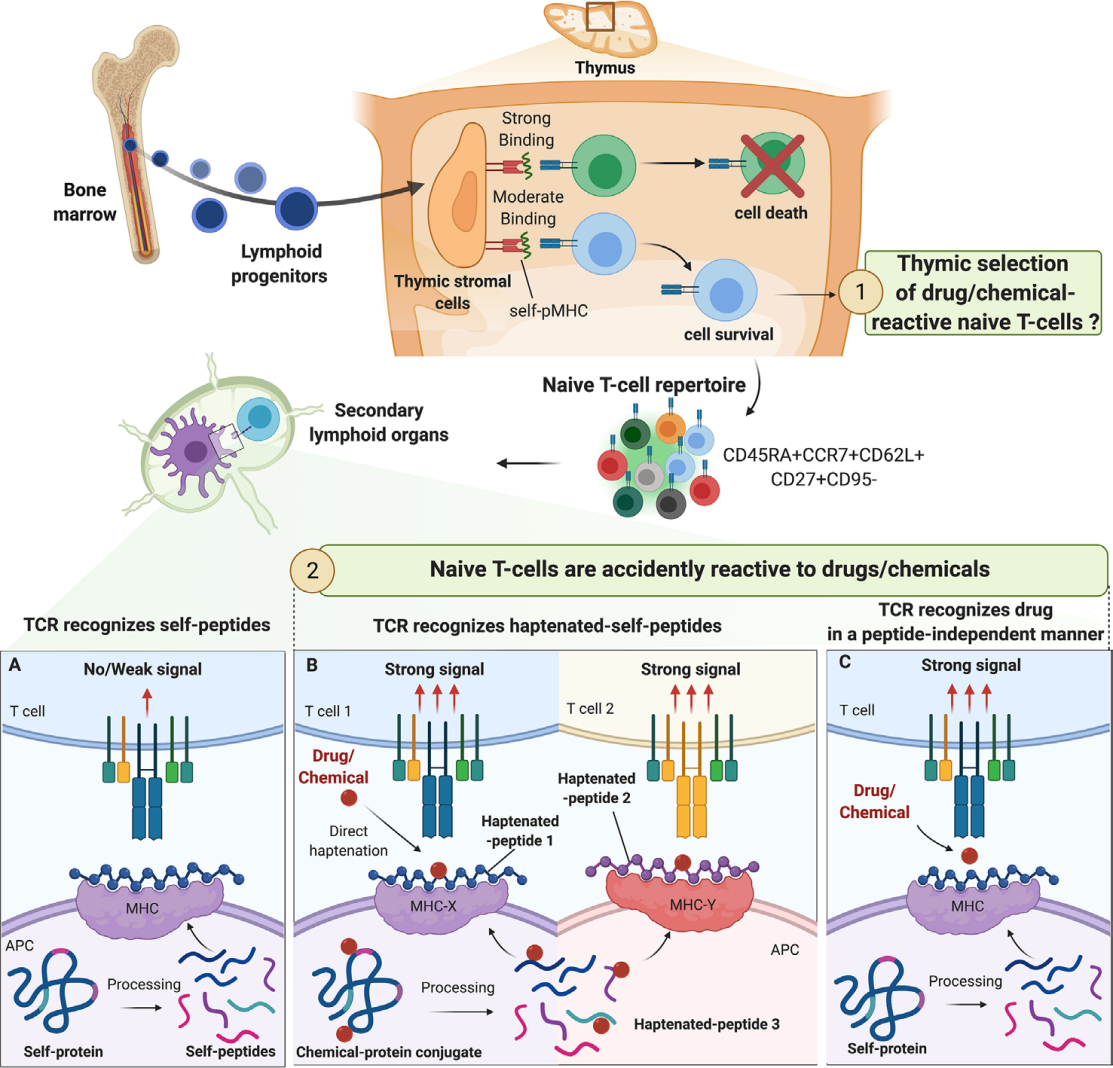
Origin of drug/chemical-reactive naive T-cell repertoire. The fate of naive T-cell precursors is dependent on the recognition of self-peptides (self-p) presented by MHC molecules on thymic stromal cells. In the periphery, naive T cells expressing CD45RA, CD62L and CCR7 constantly circulate between secondary lymphoid organs and blood in pursuit of their specific antigens. The origin of drug/chemical responsive T cells is unclear but thymic selection of drug/chemical-specific naive T cells is unlikely. The process of central selection ensures that TCRs do not bind strongly to any self-pMHC molecules in the periphery, preventing autoimmune reactions **(A)**. Drugs/chemicals may alter self-pMHC complex and haptenated self-pMHC could have a high affinity for their corresponding TCR. Depending on chemical reactivity, multiple haptenated peptides can be generated from one self-protein allowing a diversity of association with different alleles and contributing to the high prevalence of immunization/allergy observed with some drug/chemicals (beta-lactams, skin sensitizers) **(B)**. In some cases, drugs/chemicals bind to MHC proteins in a peptide-independent manner to directly activate naive T cells mimicking the conditions of alloreactivity **(C)**.

Estimation of the TCR repertoire diversity ranges from > 10^20^ (23) to 10^61^ (24, 25). Nevertheless, there are only an estimated 10^12^ T cells in the human body (26). Hence, TCR repertoire estimation vastly outnumbers the actual diversity of a person’s TCR repertoire (27). This discrepancy is explained by thymic selection where the fate of T-cell precursors is dependent on the recognition of self-peptides (self-p) presented by MHC molecules on thymic stromal cells (28). The overall outcome of the thymic selection is the maintenance of a T-cell repertoire that has sufficient, but not too strong, affinity for any self-pMHC complex (29). T cells surviving thymic selection have not yet encountered their cognate antigen, and hence are considered naive T cells (25) (**Figure 1**). Typical naive T cells express CD45RA, the co-stimulatory molecule CD27 in addition to lymph node-homing receptors CD62L and CCR7 (30). However, similar to naive T cells, human stem cell-like memory T cells (Tscm) express CD45RA, CD62L and CCR7 (31) (**Figure 1**). In this case, the expression of the death receptor CD95 that is upregulated on Tscm is taken into consideration to distinguish them from naive T cells (31). In general, Tscm constitute around 2-4% of the total T-cell population in the periphery. Due to their self-renewal and long-term persistence, Tscm were studied in autoimmunity, cancer models and HIV-1 infections. However, their implication in drug allergy is less understood (32, 33).

In the periphery, naive T cells constantly circulate between secondary lymphoid organs and blood in pursuit of their specific antigens. During their journey, the fate of naive T cells is dictated by multiple checkpoints that maintain naive T cells in quiescence (34). Upon encountering antigen, naive T cells proliferate and differentiate into activated effector T cells as well as migrate to peripheral tissues (30). A loss of thymus productivity is observed during aging. However, the human naive T-cell repertoire is maintained by peripheral T-cell proliferation driven by homeostatic factors such as IL-7 and tonic TCR signaling mediated by self-pMHC recognition (35, 36).

## How do T Cells Recognize Drugs and Chemicals?

Different studies have demonstrated that it is possible to detect drug/chemical-responding T cells in allergic patients (37–42). These T cells are activated following multiple non-mutually exclusive models, illustrating the puzzling features of TCR recognition by drugs/chemicals (43). In general, the mode of T-cell activation depends on the chemical properties of the molecule, the exposure conditions and the genetic background of the patient.

In the hapten model, drugs/chemicals or haptens bind to self-proteins to form a complex of a sufficient size to trigger an immune response. This structure is then processed by antigen-presenting cells (APCs) and the resulting haptenated peptides are presented through MHC class I or class II-dependent pathways to TCRs as *de novo* antigens (43, 44). Indeed, it is now well-accepted that MHC-restricted hapten-specific TCRs in their majority do not react to modified MHC molecules, but to haptenized peptides associated with the MHC peptide-binding groove (**Figure 1B**). Work conducted with synthetic hapten-peptide conjugates showed two major types of hapten-specific TCRs: one reacting to hapten without caring for the chemical composition of the carrier peptide, and the other contacting hapten and peptide by two apparently independent contact sites (44, 45).

In the pharmacologic interaction with immune receptors (p-i) model (**Figure 1C**), the drug binds non-covalently to either the TCR (p‐i TCR) or MHC protein (p‐i HLA) or to both in a peptide-independent manner to directly activate T cells (4, 46–49). Moreover, in the sulfamethoxazole (SMX) model of p-i TCR, molecular dynamics simulations studies showed that the drug may also bind to TCR at a position that is distant from the site of TCR-pMHC interaction, altering TCR conformation and resulting in higher affinity for self-pMHC (50). Several experimental evidence support the p-i model showing that some drugs can trigger T-cell activation without requiring intracellular antigen processing. This interaction leads to rapid T‐cell‐mediated reaction, which has features of hypersensitivity, and/or alloimmune and/or autoimmune reactions (4, 46–48).

The hapten hypothesis and the pi-concept did not provide a convincing mechanism explaining how abacavir induces adverse reactions through the activation of CD8+ T cells in a HLA-B*57:01-restricted manner (51). In this case, a new concept emerged: the altered peptide model. This model postulates that a small molecule can bind non-covalently to the MHC-binding cleft directly or in the endoplasmic reticulum (ER) and alter the specificity of peptide binding resulting in the presentation of novel peptide ligands (51–53). Using molecular dynamics simulations, recent studies demonstrated that abacavir may alter the conformational ensemble of these neo-peptides with the consequence of exposing peptide surfaces no longer recognized as self by circulating T cells (54). Peptides presented in this context are recognized as ‘‘foreign’’ by the immune system and therefore may elicit a T-cell response.

The clinical outcomes of drug or chemical allergic reactions could vary from contact dermatitis, maculopapular rashes to severe cutaneous adverse reactions and anaphylaxis, among others (1, 3, 47, 48, 55). Different T-cell recognition models can explain these multiple clinical outcomes. Contact hypersensitivity and IgE-mediated response are characterized by: 1) hapten-peptide formation, 2) dose-response effect of hapten, 3) recognition of peptide-hapten conjugates by specific TCRs and 4) rare HLA association with some allergens (13, 43, 44, 56, 57). For severe cutaneous adverse drug reactions (SCARs), the (p-i) model with drug binding to either the TCR (p-i TCR) or MHC (p-i HLA) results in T-cell activation (46). For the altered peptide repertoire model and Abacavir Hypersensitivity Syndrome, drugs bind non-covalently to regions of the HLA class I molecules within the antigen-binding cleft altering the repertoire of presented peptides and resulting in a polyclonal T-cell response (4, 52, 53, 58).

## Evidence and Characterization of a Drug/Chemical Naive T Cell Repertoire

The presence of activated and memory T cells in drug/chemical allergic patients leads to the question of the origin of these drug/chemical-responsive T cells. Since a naive T-cell repertoire is mandatory for the induction of an antigen-specific T-cell response, extensive efforts were taken to characterize drug/chemical-responsive naive T cells. T-cell priming assays provided valuable tools to detect these drug/chemical-responsive T cells (13, 59–61). Different approaches have been considered with respect to the populations of T cells and APCs used as well as the cell culture protocols and readouts (7–18, 62). Most protocols are relying on T-cell cloning performed by limiting dilutions with repetitive stimulation using APCs. Studies have tested hapten-modified dendritic cells (DCs) (12, 63) or haptenated self-proteins as an antigen source for purified naive T cells (18, 64, 65). In some protocols, regulatory T cells are removed from the co-culture system to increase the detection of weakly immunogenic drugs/chemicals (12, 60, 66). The presence of drug/chemical-responsive T cells is then detected most of the time using proliferation or cytokine production as endpoints. These approaches are not only useful to understand the mechanism of drug recognition but can also provide valuable insights for the replacement of animal testing (67). However, as expected, there are a number of problems associated with the analysis of rare antigen-specific T cells as T-cell priming assays present technical and conceptual limitations. Indeed, the high inter-donor variability limits the reliability and reproducibility of these assays. The choice of a reference protein for haptenization as well as the drug concentration used might govern the spectrum of T-cell responses. Moreover, artificial *in vitro* conditions used in these assays limit their *in vivo* relevance (67, 68).

In the early 1990s, Moulon et al. showed the ability of naive CD4+ T cells to respond to 2,4,6-trinitrobenzene sulfonic acid (TNBS), the water-soluble derivative of the contact allergen 2,4,6-trinitrochlorobenzene (TNCB) (7). These findings were further confirmed by different groups using TNBS or other chemicals (*e.g*., nickel, Bandrowski’s base, the oxidation product of p-phenylenediamine) (11, 16, 42, 61, 63, 69–72) as well as different drugs (*e.g.*, β-lactam antibiotics, SMX, dapsone, telaprevir) (18, 37, 38, 64, 65, 69, 73). Thus, the naive T-cell repertoire from every individual seems to harbor T cells able to recognize drugs and chemicals of different origins and structures. It is worth noting, that despite the presence of drug-responding T-cell repertoire in the large population, only few individuals develop allergic reactions due to additional susceptibility factors, reviewed elsewhere. (*e.g.*, HLA risk alleles, immune regulation, diseases) (55, 74, 75). Moreover, the concomitant presence of chemical-specific regulatory and effector T cells also suggests that for allergy to occur, additional signals need to be provided to break tolerance and to favor effector immune response (76).

The hapten hypothesis with binding of drug/chemical to self-proteins is the most common pathway by which chemicals (TNBS) and drugs (β-lactam and SMX) recognize and activate naive T cells (**Figure 1**). In these settings, T-cell response is dependent on (1) the presence of APCs, (2) MHC molecules, with anti‐class I and II Abs blocking their activation and (3) an intact antigen processing mechanism. However, this concept was challenged with the identification of a nickel-responding naive T-cell repertoire (11, 63, 70). Indeed, nickel, like other transitional metal ions, cannot form covalent bonds with proteins. Hence, activation of nickel-specific naive T cells may not require antigen processing as seen with classical haptens (57, 77). Instead, nickel ions form coordination complexes predominantly with nitrogen residues in histidine or arginine (57). These observations suggest that organic chemicals need to bind to MHC-associated peptides to be recognized by TCR, whereas metal ions are recognized after forming non-covalent coordination bonds with MHC molecules, bound peptides and TCR.

Beyond the simple presence of drug/chemical-responding naive T cells, the question of their frequencies in relation with the different chemical classes is also an open question. Determination of antigen specific T-cell frequency relies on different techniques. The diversity of the techniques used such as HLA class II tetramers (78–80), libraries of polyclonal expanded naive T cells followed by antigen priming (81), repeated naive T-cell priming with antigen-loaded APCs or long-term T-cell priming (16, 18, 62, 63, 65, 69, 82–85) contributed to the heterogeneity of the results. Interestingly, a good concordance was found when addressing the frequency of strong immunogens such as keyhole limpet hemocyanin (KLH)-specific T-cells with these different techniques (86). When benzylpenicillin (BP)-specific T-cell frequency was evaluated after repeated stimulation with APCs loaded with BP bioconjugates, an estimated 0.3 to 0.6 pre‐existing reactive naive T cells were detected in the blood of healthy donors per million of peripheral blood circulating CD4+ T cells (18, 65, 69). Using the same technique, 0.3 to 0.5 nickel-specific naive T cells were detected per million of circulating naive CD4+ T cells (63). These estimated frequencies can be considered very low as expected for hapten-naive specific T cells in healthy individuals (11). Surprisingly, this frequency of drugs/chemical-specific naive T cells was in the range of the one calculated for foreign antigens such as immunogenic therapeutic Abs (82), ovalbumin (82), and HIV peptide vaccine (87) but below the frequency found for KLH (18, 65, 82).

**Figure 2:**
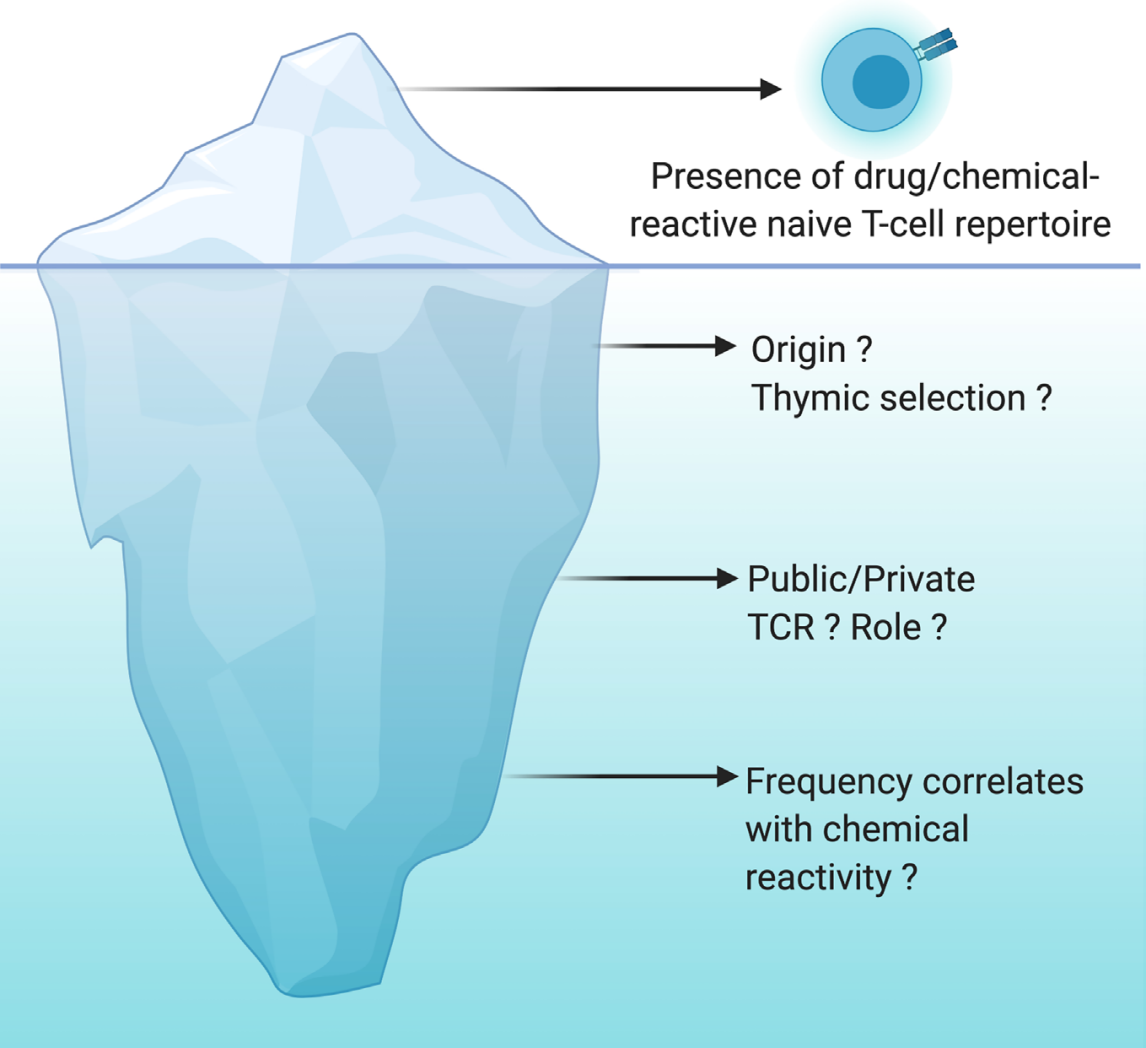
Drug/chemical-reactive naive T-cell repertoire: the tip of the iceberg. The naive T-cell repertoire of every individual harbors T cells able to recognize drugs and chemicals of different origins and structures. This concept is now well recognized and accepted but is only the tip of the iceberg. However, the origin of these T cells, the nature of their TCR (public *vs* private) as well as the correlation between their frequency and their chemical reactivity are still largely unknown. The origin of these cells is not yet clearly determined, cross-reactivity with viral antigens or specific recognition of the chemical moiety bound to a self-peptide are a working hypothesis. Do we have specific TCRs that will be more likely to expand depending on the type of recognition by TCRs (peptide-drug bioconjugates and hapten, pi-concept)? Is the frequency of naïve T cells specific for drugs and chemicals very different between individuals? Is the frequency of these T cells constant with time suggesting a constant thymic selection of T cells capable of expanding upon recognition of a drug or a chemical? All these questions are still open and are the unknown part of the iceberg.

In addition to the number of naive T cells, the composition of the naive T-cell repertoire can shape immune responses. Advances in high‐throughput sequencing technologies have enabled the detailed analysis of naive T‐cell spectrum. A private T cell response is identified when the TCR specificity toward a specific epitope is rarely observed in multiple individuals. In contrast, some other antigen-specific TCRs are frequently observed in multiple individuals and generate a public T cell response (88). For instance, nickel or SMX-responding naive T cells were driven by public TCR present in all individuals as well as by T cell Receptor Beta Variable (TRBV) genes specific for each individual (63, 89). Historically, the presence of antigen-specific public TCR was observed in a variety of infectious and autoimmune diseases and turned out to be useful for the development of vaccines and therapeutic intervention (90). Recently, Pan et al. identified a public αβTCR from the cytotoxic T cells of patients with carbamazepine-mediated SCAR and a bias for HLA-B*15:02 was also reported (91). A likely hypothesis is a pi-concept response with a public TCR recognizing a small chemical antigen presented by the preferred HLA molecule from the preexisting memory T cells. However, the cause and the role of TCR sharing within the drug/chemical-reactive naive T-cell pool of multiple individuals is still poorly addressed (**Figure 2**).

## Hypothesis for the Origin of Drug/Chemical-Responding Naive T-Cell Repertoire

Although based on a limited number of tested drugs and experiments, we can now acknowledge that most, if not all, individuals harbor a naive T‐cell repertoire for drugs and chemicals. However, while our understanding of the molecular mechanisms of TCR recognition by drugs/chemicals is expanding, the question of the mechanism driving the existence of drug/chemical-responding naive T-cell repertoire is still a challenge to be solved. Due to the artificial nature of drug-T-cell epitopes, it is unclear whether thymic selection of drug-specific T cells is a common phenomenon, remains limited to few donors, or simply does not exist. Selection of drug/chemical‐specific T cells could be a relatively rare event accounting for the low occurrence of drug/chemical allergy. On the other hand, the large T‐cell repertoire found in multiple donors underlines the potential of chemical/drug to be recognized by many donors. This latter hypothesis suggests that drugs/chemicals could be accidentally recognized by a TCR specific for another target.

Our immune system must be able to discriminate harmless non-self from dangerous non-self. There is multiple evidence that some chemicals have found very specific ways to activate the immune system by acting as danger signals (55, 64, 68, 92–102). Therefore, our immune system may have evolved to mount a specific defense mechanism avoiding prolonged exposure to reactive drugs/chemicals. Consequently, one can speculate that drug/chemical-reactive T cells are taking advantage of the imperfect central tolerance to reach the periphery and mount a protective immune response (**Figure 1**). However, there is no experimental evidence in favor of this hypothesis. It is more likely that the T-cell clones that are positively selected to recognize foreign antigens are accidently reactive with drugs or chemicals. Drugs/chemicals may alter self-pMHC complexes and haptenated self-pMHC have a high affinity for their corresponding TCR (**Figure 1B**). This concept was first elegantly described with trinitrophenyl (TNP)-reactive T cells in the context of mouse MHC-class I restricted responses (H-2K^b^) (45, 56, 72, 103, 104). This MHC class I typically harbors an octameric sequence with Phe or Tyr in position 5 and hydrophobic aliphatic amino acids in position 8 as anchors (45). As expected, this pMHC complex did not induce a strong cytotoxic T-cell response. Nevertheless, TNP-modification, mainly in position 4 of the peptide sequence, leads to CD8+ T-cell activation (45, 56). Thus, TCR recognized TNP mainly in the form of MHC-associated with haptenated peptides, and the immunodominant TNP epitopes were largely independent of the carriers’ amino acid sequence. However, how drugs/chemicals increase the affinity of pMHC to TCR is largely unknown. Haptenation of a specific amino acid could block protease-mediated enzymatic processing and/or modify the peptide-binding affinity to the transporter associated with antigen processing (TAP) (43), thereby creating structurally distinct peptide-HLA complexes. In addition, in contrast to albumin-derived peptides, BP-haptenated peptides, derived from BP-albumin conjugate, can be recognized by multiple T-cell clones and like TNP-peptides, the position of the lysine modified by BP dictates the T-cell response (18, 64). In these settings, many T cells react to haptens in a MHC-restricted but carrier-independent fashion. Thus, drug/chemical protein modification results in a particularly repetitive array of cross-reactive, immunodominant determinants that may explain the unusual strong antigenicity of these compounds (22).

Chemical reactivity may dictate the number of different proteins or residues that are haptenated. High chemical reactivity may increase the number of generated T-cell epitopes and consequently may be translated into an increase in the number of recruited naive T cells bearing different TCRs (22). Consistently, it has been shown that strong contact sensitizers induced a polyclonal T-cell response (105). Similarly, β-lactam antibiotics covalently bind to lysine residues of many proteins (20, 21, 64, 106), generating multiple binding sites on proteins and expanding the number of haptenated peptides to be recognized by T cells (22). Moreover, binding on a specific amino acid such as lysine with BP can generate more than one immunogenic epitope demonstrating that drug conjugates have some TCR specificity (43). The consequence is an augmentation of the size of the repertoire of T cells involved in β-lactam recognition.

The situation for some drugs/chemicals (*e.g.*, carbamazepine, abacavir) may be somehow more restrictive (**Figure 1C**). Indeed a specific HLA, a drug‐peptide complex and a unique TCR are the drivers of the T-cell response to certain drugs (51, 107). The most significant example is the association between abacavir hypersensitivity reaction and HLA-B*57:01 (51). Moreover, carbamazepine‐specific T cells could be primed from PBMCs of healthy human donors, carriers of both HLA‐B*15:02 and a specific TCR Vβ (108). It should be also noted that some drugs (*e.g.*, abacavir) may alter the intracellular processing of self-proteins and generate new antigenic determinants for TCRs that may not be removed from the naive T-cell repertoire during thymic selection. In these settings, naive T-cell activation may be perceived as an accident due to genetic predispositions or specific features of the molecule of concern.

Numerous patients suffering from allergic reactions are concomitantly treated for infections. Specifically, SMX-reactive T cells as well as Bandrowski’s base-responding T cells could be primed from the memory pool of healthy donors (16). Thus, one can speculate that chemical or drug naïve T-cell repertoires are mainly pathogen-specific and in some cases, these T cells may have a high propensity to cross-react with drugs or chemicals (109). Indeed, T cells responding to abacavir were also shown to recognize herpes viruses such as HSV1/2 derived-peptides (110). Moreover, carbamazepine, allopurinol, or SMX-induced DRESS can be a result of cutaneous and systemic manifestations of CD8+ T cells directed against herpes virus antigens (109, 111).

In some cases, a nonspecific inflammation, independent of chemical/drug exposure, may be sufficient to bypass the general tolerance feature of naïve T cells, irrespective of their antigen specificity. Similarly, a break in immune tolerance due to co-inhibitory molecule blockade (*e.g.*, PD‐1 and CTLA‐4) enhanced the priming of naïve T cells to drugs (74, 89). These observations were consistent with clinical studies showing increased incidence of drug hypersensitivity reactions in patients receiving immune checkpoint inhibitor therapy (112, 113). Collectively, in these different situations, the immune system may be fooled by the presence of drugs or chemicals which could lead to immunopathology.

## Concluding Remarks

It has been 30 years since the chemical-responsive naive T-cell repertoire was first described. Since then, multiple examples have been documented and there is no doubt that more will be uncovered. Not surprising anymore that drugs/chemicals, in their majority, seem to be recognized by the same molecular mechanisms as protein antigens. However, whether thymic selection of drug/chemical-specific T cells is a common phenomenon, remains limited to few donors, or simply does not exist is still unclear (**Figure 2**). If the naive T-cell repertoire contributes to drug/chemical allergy, then it is plausible that these reactions stem from *de novo* responses to the drug/chemical, where these specific T cells took advantage of the imperfect central tolerance to reach the periphery and mount a protective immune response. Yet, it is more likely that naive T cells are accidently reactive to a drug or a chemical. Several studies support the theory that chemical modification of self-proteins increases the affinity of self-pMHC to their cognate TCR or results in new antigenic determinants (**Figure 1**). Good progress has also been made in our mechanistic understanding of TCR recognition of drugs and chemicals (13, 43). However, less is known about the origin of these T cells, the nature of their TCR (public *vs* private) as well as the correlation between their frequency and the chemical reactivity (**Figure 2**). Answering these questions can be expected to open up new and exciting avenues for drug/chemical allergy.

## Author Contributions

RB, AF, and MP wrote the manuscript. All authors contributed to the article and approved the submitted version.

## Conflict of Interest

The authors declare that the research was conducted in the absence of any commercial or financial relationships that could be construed as a potential conflict of interest.

## References

[1] Hammond S Thomson PJ Ogese MO Naisbitt DJ. T-Cell Activation by Low Molecular Weight Drugs and Factors That Influence Susceptibility to Drug Hypersensitivity. Chem Res Toxicol (2020) 33(1):77–94. 10.1021/acs.chemrestox.9b00327 31687800

[2] Vocanson M Naisbitt DJ Nicolas JF. Current Perspective of the Etiopathogenesis of Delayed-Type, and T-Cell-Mediated Drug-Related Skin Diseases. J Allergy Clin Immunol (2020) 145(4):1142–4. 10.1016/j.jaci.2020.01.030 32018033

[3] Wheatley LM Plaut M Schwaninger JM Banerji A Castells M Finkelman FD. Report From the National Institute of Allergy and Infectious Diseases Workshop on Drug Allergy. J Allergy Clin Immunol (2015) 136(2):262–71.e2. 10.1016/j.jaci.2015.05.027 26254053 PMC4529958

[4] Pichler WJ Naisbitt DJ Park BK. Immune Pathomechanism of Drug Hypersensitivity Reactions. J Allergy Clin Immunol (2011) 127(3 Suppl):S74–81. 10.1016/j.jaci.2010.11.048 21354503

[5] Amali MO Sullivan A Jenkins RE Farrell J Meng X Faulkner L. Detection of Drug-Responsive B Lymphocytes and Antidrug Igg in Patients With Beta-Lactam Hypersensitivity. Allergy (2017) 72(6):896–907. 10.1111/all.13087 27861994

[6] Rozieres A Vocanson M Said BB Nosbaum A Nicolas JF. Role of T Cells in Nonimmediate Allergic Drug Reactions. Curr Opin Allergy Clin Immunol (2009) 9(4):305–10. 10.1097/ACI.0b013e32832d565c 19474707

[7] Moulon C Peguet-Navarro J Courtellemont P Redziniak G Schmitt D. *In Vitro* Primary Sensitization and Restimulation of Hapten-Specific T Cells by Fresh and Cultured Human Epidermal Langerhans’ Cells. Immunology (1993) 80(3):373–9. PMC1422213 7507088

[8] Krasteva M Peguet-Navarro J Moulon C Courtellemont P Redziniak G Schmitt D. *In Vitro* Primary Sensitization of Hapten-Specific T Cells by Cultured Human Epidermal Langerhans Cells–A Screening Predictive Assay for Contact Sensitizers. Clin Exp Allergy (1996) 26(5):563–70. 10.1046/j.1365-2222.1996.d01-342.x 8735869

[9] Dai R Streilein JW. Naive, Hapten-Specific Human T Lymphocytes Are Primed *In Vitro* With Derivatized Blood Mononuclear Cells. J Invest Dermatol (1998) 110(1):29–33. 10.1046/j.1523-1747.1998.00088.x 9424083

[10] Rougier N Redziniak G Schmitt D Vincent C. Evaluation of the Capacity of Dendritic Cells Derived From Cord Blood CD34+ Precursors to Present Haptens to Unsensitized Autologous T Cells *In Vitro* . J Invest Dermatol (1998) 110(4):348–52. 10.1046/j.1523-1747.1998.00150.x 9540973

[11] Rustemeyer T De Ligter S Von Blomberg BM Frosch PJ Scheper RJ. Human T Lymphocyte Priming *In Vitro* by Haptenated Autologous Dendritic Cells. Clin Exp Immunol (1999) 117(2):209–16. 10.1046/j.1365-2249.1999.00958.x PMC1905350 10444249

[12] Vocanson M Cluzel-Tailhardat M Poyet G Valeyrie M Chavagnac C Levarlet B. Depletion of Human Peripheral Blood Lymphocytes in CD25+ Cells Allows for the Sensitive *In Vitro* Screening of Contact Allergens. J Invest Dermatol (2008) 128(8):2119–22. 10.1038/jid.2008.15 18256693

[13] Martin SF Esser PR Schmucker S Dietz L Naisbitt DJ Park BK. T-Cell Recognition of Chemicals, Protein Allergens and Drugs: Towards the Development of *In Vitro* Assays. Cell Mol Life Sci (2010) 67(24):4171–84. 10.1007/s00018-010-0495-3 PMC11115584 20717835

[14] El-Ghaiesh S Monshi MM Whitaker P Jenkins R Meng X Farrell J. Characterization of the Antigen Specificity of T-Cell Clones From Piperacillin-Hypersensitive Patients With Cystic Fibrosis. J Pharmacol Exp Ther (2012) 341(3):597–610. 10.1124/jpet.111.190900 22371438 PMC3362878

[15] Naisbitt DJ Nattrass RG Ogese MO. *In Vitro* Diagnosis of Delayed-Type Drug Hypersensitivity: Mechanistic Aspects and Unmet Needs. Immunol Allergy Clin North Am (2014) 34(3):691–705. 10.1016/j.iac.2014.04.009 25017686

[16] Gibson A Kim SH Faulkner L Evely J Pirmohamed M Park KB. *In Vitro* Priming of Naive T-Cells With P-Phenylenediamine and Bandrowski’s Base. Chem Res Toxicol (2015) 28(10):2069–77. 10.1021/acs.chemrestox.5b00294 26355666

[17] Moed H von Blomberg M Bruynzeel DP Scheper R Gibbs S Rustemeyer T. Improved Detection of Allergen-Specific T-Cell Responses in Allergic Contact Dermatitis Through the Addition of ‘Cytokine Cocktails’. Exp Dermatol (2005) 14(8):634–40. 10.1111/j.0906-6705.2005.00344.x 16026586

[18] Azoury ME Fili L Bechara R Scornet N de Chaisemartin L Weaver RJ. Identification of T-Cell Epitopes From Benzylpenicillin Conjugated to Human Serum Albumin and Implication in Penicillin Allergy. Allergy (2018) 73(8):1662–72. 10.1111/all.13418 29355985

[19] Castrejon JL Berry N El-Ghaiesh S Gerber B Pichler WJ Park BK. Stimulation of Human T Cells With Sulfonamides and Sulfonamide Metabolites. J Allergy Clin Immunol (2010) 125(2):411–8.e4. 10.1016/j.jaci.2009.10.031 20159253

[20] Meng X Al-Attar Z Yaseen FS Jenkins R Earnshaw C Whitaker P. Definition of the Nature and Hapten Threshold of the Beta-Lactam Antigen Required for T Cell Activation *In Vitro* and in Patients. J Immunol (2017) 198(11):4217–27. 10.4049/jimmunol.1700209 PMC5444528 28438900

[21] Whitaker P Meng X Lavergne SN El-Ghaiesh S Monshi M Earnshaw C. Mass Spectrometric Characterization of Circulating and Functional Antigens Derived From Piperacillin in Patients With Cystic Fibrosis. J Immunol (2011) 187(1):200–11. 10.4049/jimmunol.1100647 PMC3145118 21606251

[22] Esser PR Kimber I Martin SF. Correlation of Contact Sensitizer Potency With T Cell Frequency and TCR Repertoire Diversity. Exp Suppl (2014) 104:101–14. 10.1007/978-3-0348-0726-5_8 24214621

[23] Zarnitsyna VI Evavold BD Schoettle LN Blattman JN Antia R. Estimating the Diversity, Completeness, and Cross-Reactivity of the T Cell Repertoire. Front Immunol (2013) 4:485. 10.3389/fimmu.2013.00485 24421780 PMC3872652

[24] Das J Jayaprakash C. Systems Immunology: An Introduction to Modeling Methods for Scientists. In: Foundations of Biochemistry and Biophysics. Boca Raton, FL: CRC Press, Taylor and Francis Group (2019). p. 1.

[25] de Greef PC Oakes T Gerritsen B Ismail M Heather JM Hermsen R. The Naive T-Cell Receptor Repertoire has an Extremely Broad Distribution of Clone Sizes. Elife (2020) 9:e49900. 10.7554/eLife.49900 32187010 PMC7080410

[26] Arstila TP Casrouge A Baron V Even J Kanellopoulos J Kourilsky P. A Direct Estimate of the Human Alphabeta T Cell Receptor Diversity. Science (1999) 286(5441):958–61. 10.1126/science.286.5441.958 10542151

[27] Laydon DJ Bangham CR Asquith B. Estimating T-Cell Repertoire Diversity: Limitations of Classical Estimators and a New Approach. Philos Trans R Soc Lond B Biol Sci (2015) 370(1675):20140291. 10.1098/rstb.2014.0291 26150657 PMC4528489

[28] Klein L Kyewski B Allen PM Hogquist KA. Positive and Negative Selection of the T Cell Repertoire: What Thymocytes See (and Don’t See). Nat Rev Immunol (2014) 14(6):377–91. 10.1038/nri3667 PMC4757912 24830344

[29] McDonald BD Bunker JJ Erickson SA Oh-Hora M Bendelac A. Crossreactive Alphabeta T Cell Receptors Are the Predominant Targets of Thymocyte Negative Selection. Immunity (2015) 43(5):859–69. 10.1016/j.immuni.2015.09.009 PMC4654978 26522985

[30] van den Broek T Borghans JAM van Wijk F. The Full Spectrum of Human Naive T Cells. Nat Rev Immunol (2018) 18(6):363–73. 10.1038/s41577-018-0001-y 29520044

[31] Gattinoni L Lugli E Ji Y Pos Z Paulos CM Quigley MF. A Human Memory T Cell Subset With Stem Cell-Like Properties. Nat Med (2011) 17(10):1290–7. 10.1038/nm.2446 PMC3192229 21926977

[32] Flynn JK Gorry PR. Stem Memory T Cells (TSCM)-Their Role in Cancer and HIV Immunotherapies. Clin Transl Immunol (2014) 3(7):e20. 10.1038/cti.2014.16 PMC4232066 25505968

[33] Gattinoni L Speiser DE Lichterfeld M Bonini C. T Memory Stem Cells in Health and Disease. Nat Med (2017) 23(1):18–27. 10.1038/nm.4241 28060797 PMC6354775

[34] ElTanbouly MA Noelle RJ. Rethinking Peripheral T Cell Tolerance: Checkpoints Across a T Cell’s Journey. Nat Rev Immunol (2020) 21(4):257–67. 10.1038/s41577-020-00454-2 PMC12536352 33077935

[35] Egorov ES Kasatskaya SA Zubov VN Izraelson M Nakonechnaya TO Staroverov DB. The Changing Landscape of Naive T Cell Receptor Repertoire With Human Aging. Front Immunol (2018) 9:1618. 10.3389/fimmu.2018.01618 30087674 PMC6066563

[36] Velardi E Tsai JJ van den Brink MRM. T Cell Regeneration After Immunological Injury. Nat Rev Immunol (2020) 21(5):277–91. 10.1038/s41577-020-00457-z PMC7583557 33097917

[37] Yaseen FS Yaseen FS Saide K Kim SH Monshi M Tailor A Wood S. Promiscuous T-Cell Responses to Drugs and Drug-Haptens. J Allergy Clin Immunol (2015) 136(2):474–6.e8. 10.1016/j.jaci.2015.02.036 25910715

[38] Zhao Q Alhilali K Alzahrani A Almutairi M Amjad J Liu H. Dapsone- and Nitroso Dapsone-Specific Activation of T Cells From Hypersensitive Patients Expressing the Risk Allele HLA-B*13:01. Allergy (2019) 74(8):1533–48. 10.1111/all.13769 PMC6767778 30844087

[39] Sullivan A Wang E Farrell J Whitaker P Faulkner L Peckham D. Beta-Lactam Hypersensitivity Involves Expansion of Circulating and Skin-Resident TH22 Cells. J Allergy Clin Immunol (2018) 141(1):235–49.e8. 10.1016/j.jaci.2017.01.020 28219704

[40] Meng X Earnshaw CJ Tailor A Jenkins RE Waddington JC Whitaker P. Amoxicillin and Clavulanate Form Chemically and Immunologically Distinct Multiple Haptenic Structures in Patients. Chem Res Toxicol (2016) 29(10):1762–72. 10.1021/acs.chemrestox.6b00253 27603302

[41] Usui T Whitaker P Meng X Watson J Antoine DJ French NS. Detection of Drug-Responsive T-Lymphocytes in a Case of Fatal Antituberculosis Drug-Related Liver Injury. Chem Res Toxicol (2016) 29(11):1793–5. 10.1021/acs.chemrestox.6b00393 27933861

[42] Cavani A Mei D Guerra E Corinti S Giani M Pirrotta L. Patients With Allergic Contact Dermatitis to Nickel and Nonallergic Individuals Display Different Nickel-Specific T Cell Responses. Evidence for the Presence of Effector CD8+ and Regulatory CD4+ T Cells. J Invest Dermatol (1998) 111(4):621–8. 10.1046/j.1523-1747.1998.00334.x 9764843

[43] Meng X Yerly D Naisbitt DJ. Mechanisms Leading to T-Cell Activation in Drug Hypersensitivity. Curr Opin Allergy Clin Immunol (2018) 18(4):317–24. 10.1097/ACI.0000000000000458 29905574

[44] Earnshaw CJ Pecaric-Petkovic T Park BK Naisbitt DJ. T Cell Responses to Drugs and Drug Metabolites. Exp Suppl (2014) 104:137–63. 10.1007/978-3-0348-0726-5_10 24214623

[45] Weltzien HU Moulon C Martin S Padovan E Hartmann U Kohler J. T Cell Immune Responses to Haptens. Structural Models for Allergic and Autoimmune Reactions. Toxicology (1996) 107(2):141–51. 10.1016/0300-483X(95)03253-C 8599173

[46] Pichler WJ Adam J Watkins S Wuillemin N Yun J Yerly D. Drug Hypersensitivity: How Drugs Stimulate T Cells *via* Pharmacological Interaction With Immune Receptors. Int Arch Allergy Immunol (2015) 168(1):13–24. 10.1159/000441280 26524432

[47] Pichler WJ Hausmann O. Classification of Drug Hypersensitivity Into Allergic, P-I, and Pseudo-Allergic Forms. Int Arch Allergy Immunol (2016) 171(3-4):166–79. 10.1159/000453265 27960170

[48] Pichler WJ. Immune Pathomechanism and Classification of Drug Hypersensitivity. Allergy (2019) 74(8):1457–71. 10.1111/all.13765 30843233

[49] Zanni MP von Greyerz S Schnyder B Brander KA Frutig K Hari Y. HLA-Restricted, Processing- and Metabolism-Independent Pathway of Drug Recognition by Human Alpha Beta T Lymphocytes. J Clin Invest (1998) 102(8):1591–8. 10.1172/JCI3544 PMC509010 9788973

[50] Watkins S Pichler WJ. Sulfamethoxazole Induces a Switch Mechanism in T Cell Receptors Containing Tcrvbeta20-1, Altering Phla Recognition. PLoS One (2013) 8(10):e76211. 10.1371/journal.pone.0076211 24116097 PMC3792127

[51] Mallal S Nolan D Witt C Masel G Martin AM Moore C. Association Between Presence of HLA-B*5701, HLA-DR7, and HLA-DQ3 and Hypersensitivity to HIV-1 Reverse-Transcriptase Inhibitor Abacavir. Lancet (2002) 359(9308):727–32. 10.1016/S0140-6736(02)07873-X 11888582

[52] Illing PT Vivian JP Dudek NL Kostenko L Chen Z Bharadwaj M. Immune Self-Reactivity Triggered by Drug-Modified HLA-Peptide Repertoire. Nature (2012) 486(7404):554–8. 10.1038/nature11147 22722860

[53] Ostrov DA Grant BJ Pompeu YA Sidney J Harndahl M Southwood S. Drug Hypersensitivity Caused by Alteration of the MHC-Presented Self-Peptide Repertoire. Proc Natl Acad Sci USA (2012) 109(25):9959–64. 10.1073/pnas.1207934109 PMC3382472 22645359

[54] Fodor J Riley BT Kass I Buckle AM Borg NA. The Role of Conformational Dynamics in Abacavir-Induced Hypersensitivity Syndrome. Sci Rep (2019) 9(1):10523. 10.1038/s41598-019-47001-1 31324847 PMC6642150

[55] Pallardy M Bechara R. Chemical or Drug Hypersensitivity: Is the Immune System Clearing the Danger? Toxicol Sci (2017) 158(1):14–22. 10.1093/toxsci/kfx084 28472426

[56] Martin S Ortmann B Pflugfelder U Birsner U Weltzien HU. Role of Hapten-Anchoring Peptides in Defining Hapten-Epitopes for MHC-Restricted Cytotoxic T Cells. Cross-Reactive TNP-Determinants on Different Peptides. J Immunol (1992) 149(8):2569–75. 1383319

[57] Weltzien HU Martin SF Nicolas JF. T Cell Responses to Contact Allergens. Exp Suppl (2014) 104:41–9. 10.1007/978-3-0348-0726-5_4 24214617

[58] Adam J Eriksson KK Schnyder B Fontana S Pichler WJ Yerly D. Avidity Determines T-Cell Reactivity in Abacavir Hypersensitivity. Eur J Immunol (2012) 42(7):1706–16. 10.1002/eji.201142159 22585534

[59] Usui T Faulkner L Farrell J French NS Alfirevic A Pirmohamed M. Application of *In Vitro* T Cell Assay Using Human Leukocyte Antigen-Typed Healthy Donors for the Assessment of Drug Immunogenicity. Chem Res Toxicol (2018) 31(3):165–7. 10.1021/acs.chemrestox.8b00030 29436218

[60] Ogese MO Watkinson J Lister A Faulkner L Gibson A Hillegas A. Development of an Improved T-Cell Assay to Assess the Intrinsic Immunogenicity of Haptenic Compounds. Toxicol Sci (2020) 175(2):266–78. 10.1093/toxsci/kfaa034 32159798

[61] Dietz L Esser PR Schmucker SS Goette I Richter A Schnolzer M. Tracking Human Contact Allergens: From Mass Spectrometric Identification of Peptide-Bound Reactive Small Chemicals to Chemical-Specific Naive Human T-Cell Priming. Toxicol Sci (2010) 117(2):336–47. 10.1093/toxsci/kfq209 20631061

[62] Faulkner L Gibson A Sullivan A Tailor A Usui T Alfirevic A. Detection of Primary T Cell Responses to Drugs and Chemicals in HLA-Typed Volunteers: Implications for the Prediction of Drug Immunogenicity. Toxicol Sci (2016) 154(2):416–29. 10.1093/toxsci/kfw177 27637899

[63] Bechara R Pollastro S Azoury ME Szely N Maillere B de Vries N. Identification and Characterization of Circulating Naive CD4+ and CD8+ T Cells Recognizing Nickel. Front Immunol (2019) 10:1331. 10.3389/fimmu.2019.01331 31249573 PMC6582854

[64] Scornet N Delarue-Cochin S Azoury ME Le Mignon M Chemelle JA Nony E. Bioinspired Design and Oriented Synthesis of Immunogenic Site-Specifically Penicilloylated Peptides. Bioconjug Chem (2016) 27(11):2629–45. 10.1021/acs.bioconjchem.6b00393 27552359

[65] Nhim C Delluc S Halgand F de Chaisemartin L Weaver RJ Claude N. Identification and Frequency of Circulating CD4(+) T Lymphocytes Specific to Benzylpenicillin in Healthy Donors. Allergy (2013) 68(7):899–905. 10.1111/all.12173 23751122

[66] Curotto de Lafaille MA Lafaille JJ. Natural and Adaptive Foxp3+ Regulatory T Cells: More of the Same or a Division of Labor? Immunity (2009) 30(5):626–35. 10.1016/j.immuni.2009.05.002 19464985

[67] Martin SF Schmucker SS Richter A. Tools and Methods for Identification and Analysis of Rare Antigen-Specific T Lymphocytes. Exp Suppl (2014) 104:73–88. 10.1007/978-3-0348-0726-5_6 24214619

[68] van Vliet E Kuhnl J Goebel C Martinozzi-Teissier S Alepee N Ashikaga T. State-of-the-Art and New Options to Assess T Cell Activation by Skin Sensitizers: Cosmetics Europe Workshop. ALTEX (2018) 35(2):179–92. 10.14573/altex.1709011 28968481

[69] Bechara R Maillere B Joseph D Weaver RJ Pallardy M. Identification and Characterization of a Naive CD8+ T Cell Repertoire for Benzylpenicillin. Clin Exp Allergy (2019) 49(5):636–43. 10.1111/cea.13338 30657219

[70] Lisby S Hansen LH Menn T Baadsgaard O. Nickel-Induced Proliferation of Both Memory and Naive T Cells in Patch Test-Negative Individuals. Clin Exp Immunol (1999) 117(2):217–22. 10.1046/j.1365-2249.1999.00967.x PMC1905330 10444250

[71] Cavani A Nasorri F Ottaviani C Sebastiani S De Pita O Girolomoni G. Human CD25+ Regulatory T Cells Maintain Immune Tolerance to Nickel in Healthy, Nonallergic Individuals. J Immunol (2003) 171(11):5760–8. 10.4049/jimmunol.171.11.5760 14634084

[72] Moulon C Vollmer J Weltzien HU. Characterization of Processing Requirements and Metal Cross-Reactivities in T Cell Clones From Patients With Allergic Contact Dermatitis to Nickel. Eur J Immunol (1995) 25(12):3308–15. 10.1002/eji.1830251216 8566016

[73] Alhilali KA Al-Attar Z Gibson A Tailor A Meng X Monshouwer M. Characterization of Healthy Donor-Derived T-Cell Responses Specific to Telaprevir Diastereomers. Toxicol Sci (2019) 168(2):597–609. 10.1093/toxsci/kfz007 30649540

[74] Naisbitt DJ Olsson-Brown A Gibson A Meng X Ogese MO Tailor A. Immune Dysregulation Increases the Incidence of Delayed-Type Drug Hypersensitivity Reactions. Allergy (2020) 75(4):781–97. 10.1111/all.14127 31758810

[75] Gibson A Ogese M Pirmohamed M. Genetic and Nongenetic Factors That May Predispose Individuals to Allergic Drug Reactions. Curr Opin Allergy Clin Immunol (2018) 18(4):325–32. 10.1097/ACI.0000000000000459 29889140

[76] Cavani A. Breaking Tolerance to Nickel. Toxicology (2005) 209(2):119–21. 10.1016/j.tox.2004.12.021 15767023

[77] Thierse HJ Gamerdinger K Junkes C Guerreiro N Weltzien HU. T Cell Receptor (TCR) Interaction With Haptens: Metal Ions as Non-Classical Haptens. Toxicology (2005) 209(2):101–7. 10.1016/j.tox.2004.12.015 15767020

[78] Obar JJ Khanna KM Lefrancois L. Endogenous Naive CD8+ T Cell Precursor Frequency Regulates Primary and Memory Responses to Infection. Immunity (2008) 28(6):859–69. 10.1016/j.immuni.2008.04.010 PMC2836785 18499487

[79] Hataye J Moon JJ Khoruts A Reilly C Jenkins MK. Naive and Memory CD4+ T Cell Survival Controlled by Clonal Abundance. Science (2006) 312(5770):114–6. 10.1126/science.1124228 16513943

[80] Moon JJ Chu HH Pepper M McSorley SJ Jameson SC Kedl RM. Naive CD4(+) T Cell Frequency Varies for Different Epitopes and Predicts Repertoire Diversity and Response Magnitude. Immunity (2007) 27(2):203–13. 10.1016/j.immuni.2007.07.007 PMC2200089 17707129

[81] Geiger R Duhen T Lanzavecchia A Sallusto F. Human Naive and Memory CD4+ T Cell Repertoires Specific for Naturally Processed Antigens Analyzed Using Libraries of Amplified T Cells. J Exp Med (2009) 206(7):1525–34. 10.1084/jem.20090504 PMC2715094 19564353

[82] Delluc S Ravot G Maillere B. Quantitative Analysis of the CD4 T-Cell Repertoire Specific to Therapeutic Antibodies in Healthy Donors. FASEB J (2011) 25(6):2040–8. 10.1096/fj.10-173872 21368101

[83] Meunier S Menier C Marcon E Lacroix-Desmazes S Maillere B. CD4 T Cells Specific for Factor VIII Are Present at High Frequency in Healthy Donors and Comprise Naive and Memory Cells. Blood Adv (2017) 1(21):1842–7. 10.1182/bloodadvances.2017008706 PMC5728100 29296830

[84] Azam A Gallais Y Mallart S Illiano S Duclos O Prades C. Healthy Donors Exhibit a CD4 T Cell Repertoire Specific to the Immunogenic Human Hormone H2-Relaxin Before Injection. J Immunol (2019) 202(12):3507–13. 10.4049/jimmunol.1800856 31101669

[85] Meunier S de Bourayne M Hamze M Azam A Correia E Menier C. Specificity of the T Cell Response to Protein Biopharmaceuticals. Front Immunol (2020) 11:1550. 10.3389/fimmu.2020.01550 32793213 PMC7387651

[86] Maillere B. Comment on “The Role of Naive T Cell Precursor Frequency and Recruitment in Dictating Immune Response Magnitude”. J Immunol (2013) 190(5):1895. 10.4049/jimmunol.1290079 23417524

[87] Castelli FA Szely N Olivain A Casartelli N Grygar C Schneider A. Hierarchy of CD4 T Cell Epitopes of the ANRS Lipo5 Synthetic Vaccine Relies on the Frequencies of Pre-Existing Peptide-Specific T Cells in Healthy Donors. J Immunol (2013) 190(11):5757–63. 10.4049/jimmunol.1300145 23636059

[88] Venturi V Price DA Douek DC Davenport MP. The Molecular Basis for Public T-Cell Responses? Nat Rev Immunol (2008) 8(3):231–8. 10.1038/nri2260 18301425

[89] Gibson A Faulkner L Lichtenfels M Ogese M Al-Attar Z Alfirevic A. The Effect of Inhibitory Signals on the Priming of Drug Hapten-Specific T Cells That Express Distinct Vbeta Receptors. J Immunol (2017) 199(4):1223–37. 10.4049/jimmunol.1602029 PMC5551967 28687658

[90] Li H Ye C Ji G Han J. Determinants of Public T Cell Responses. Cell Res (2012) 22(1):33–42. 10.1038/cr.2012.1 22212481 PMC3351923

[91] Pan RY Chu MT Wang CW Lee YS Lemonnier F Michels AW. Identification of Drug-Specific Public TCR Driving Severe Cutaneous Adverse Reactions. Nat Commun (2019) 10(1):3569. 10.1038/s41467-019-11396-2 31395875 PMC6687717

[92] Antonios D Rousseau P Larange A Kerdine-Romer S Pallardy M. Mechanisms of IL-12 Synthesis by Human Dendritic Cells Treated With the Chemical Sensitizer Niso4. J Immunol (2010) 185(1):89–98. 10.4049/jimmunol.0901992 20525893

[93] Bechara R Antonios D Azouri H Pallardy M. Nickel Sulfate Promotes IL-17A Producing CD4+ T Cells by an IL-23-Dependent Mechanism Regulated by TLR4 and Jak-STAT Pathways. J Invest Dermatol (2017) 137(10):2140–8. 10.1016/j.jid.2017.05.025 28634033

[94] Raffalli C Clouet E Kuresepi S Damiens MH Lepoittevin JP Pallardy M. Editor’s Highlight: Fragrance Allergens Linalool and Limonene Allylic Hydroperoxides in Skin Allergy: Mechanisms of Action Focusing on Transcription Factor Nrf2. Toxicol Sci (2018) 161(1):139–48. 10.1093/toxsci/kfx207 29029310

[95] Sanderson JP Naisbitt DJ Farrell J Ashby CA Tucker MJ Rieder MJ. Sulfamethoxazole and its Metabolite Nitroso Sulfamethoxazole Stimulate Dendritic Cell Costimulatory Signaling. J Immunol (2007) 178(9):5533–42. 10.4049/jimmunol.178.9.5533 17442935

[96] Zhang X Sharma AM Uetrecht J. Identification of Danger Signals in Nevirapine-Induced Skin Rash. Chem Res Toxicol (2013) 26(9):1378–83. 10.1021/tx400232s 23947594

[97] Elzagallaai AA Sultan EA Bend JR Abuzgaia AM Loubani E Rieder MJ. Role of Oxidative Stress in Hypersensitivity Reactions to Sulfonamides. J Clin Pharmacol (2020) 60(3):409–21. 10.1002/jcph.1535 31709574

[98] Schmidt M Raghavan B Muller V Vogl T Fejer G Tchaptchet S. Crucial Role for Human Toll-Like Receptor 4 in the Development of Contact Allergy to Nickel. Nat Immunol (2010) 11(9):814–9. 10.1038/ni.1919 20711192

[99] Rachmawati D Bontkes HJ Verstege MI Muris J von Blomberg BM Scheper RJ. Transition Metal Sensing by Toll-Like Receptor-4: Next to Nickel, Cobalt and Palladium Are Potent Human Dendritic Cell Stimulators. Contact Dermatitis (2013) 68(6):331–8. 10.1111/cod.12042 23692033

[100] Lopez S Gomez E Torres MJ Pozo D Fernandez TD Ariza A. Betalactam Antibiotics Affect Human Dendritic Cells Maturation Through MAPK/NF-Kb Systems. Role in Allergic Reactions to Drugs. Toxicol Appl Pharmacol (2015) 288(3):289–99. 10.1016/j.taap.2015.08.001 26254762

[101] Martin SF Dudda JC Bachtanian E Lembo A Liller S Durr C. Toll-Like Receptor and IL-12 Signaling Control Susceptibility to Contact Hypersensitivity. J Exp Med (2008) 205(9):2151–62. 10.1084/jem.20070509 PMC2526208 18725520

[102] Esser PR Wolfle U Durr C von Loewenich FD Schempp CM Freudenberg MA. Contact Sensitizers Induce Skin Inflammation *via* ROS Production and Hyaluronic Acid Degradation. PLoS One (2012) 7(7):e41340. 10.1371/journal.pone.0041340 22848468 PMC3405137

[103] Martin S Weltzien HU. T Cell Recognition of Haptens, a Molecular View. Int Arch Allergy Immunol (1994) 104(1):10–6. 10.1159/000236703 7524836

[104] Kohler J Hartmann U Grimm R Pflugfelder U Weltzien HU. Carrier-Independent Hapten Recognition and Promiscuous MHC Restriction by CD4 T Cells Induced by Trinitrophenylated Peptides. J Immunol (1997) 158(2):591–7. 8992972

[105] Martin S Delattre V Leicht C Weltzien HU Simon JC. A High Frequency of Allergen-Specific CD8+ Tc1 Cells Is Associated With the Murine Immune Response to the Contact Sensitizer Trinitrophenyl. Exp Dermatol (2003) 12(1):78–85. 10.1034/j.1600-0625.2003.120110.x 12631250

[106] Jenkins RE Meng X Elliott VL Kitteringham NR Pirmohamed M Park BK. Characterisation of Flucloxacillin and 5-Hydroxymethyl Flucloxacillin Haptenated HSA *In Vitro* and *In Vivo* . Proteomics Clin Appl (2009) 3(6):720–9. 10.1002/prca.200800222 21136982

[107] Adam J Wuillemin N Watkins S Jamin H Eriksson KK Villiger P. Abacavir Induced T Cell Reactivity From Drug Naive Individuals Shares Features of Allo-Immune Responses. PLoS One (2014) 9(4):e95339. 10.1371/journal.pone.0095339 24751900 PMC3994040

[108] Ko TM Chung WH Wei CY Shih HY Chen JK Lin CH. Shared and Restricted T-Cell Receptor Use is Crucial for Carbamazepine-Induced Stevens-Johnson Syndrome. J Allergy Clin Immunol (2011) 128(6):1266–76.e11. 10.1016/j.jaci.2011.08.013 21924464

[109] White KD Chung WH Hung SI Mallal S Phillips EJ. Evolving Models of the Immunopathogenesis of T Cell-Mediated Drug Allergy: The Role of Host, Pathogens, and Drug Response. J Allergy Clin Immunol (2015) 136(2):219–34; quiz 235. 10.1016/j.jaci.2015.05.050 PMC4577472 26254049

[110] Yerly D Pompeu YA Schutte RJ Eriksson KK Strhyn A Bracey AW. Structural Elements Recognized by Abacavir-Induced T Cells. Int J Mol Sci (2017) 18(7):1464. 10.3390/ijms18071464 PMC5535955 28686208

[111] Picard D Janela B Descamps V D'Incan M Courville P Jacquot S. Drug Reaction With Eosinophilia and Systemic Symptoms (DRESS): A Multiorgan Antiviral T Cell Response. Sci Transl Med (2010) 2(46):46ra62. 10.1126/scitranslmed.3001116 20739682

[112] Ford M Sahbudin I Filer A Steven N Fisher BA. High Proportion of Drug Hypersensitivity Reactions to Sulfasalazine Following its Use in Anti-PD-1-Associated Inflammatory Arthritis. Rheumatol (Oxf) (2018) 57(12):2244–6. 10.1093/rheumatology/key234 30107548

[113] Phillips GS Wu J Hellmann MD Postow MA Rizvi NA Freites-Martinez A. Treatment Outcomes of Immune-Related Cutaneous Adverse Events. J Clin Oncol (2019) 37(30):2746–58. 10.1200/JCO.18.02141 PMC7001790 31216228

